# Expanding the substrates for a bacterial hydrogenlyase reaction

**DOI:** 10.1099/mic.0.000471

**Published:** 2017-05-10

**Authors:** Ciaran M Lamont, Ciarán L Kelly, Constanze Pinske, Grant Buchanan, Tracy Palmer, Frank Sargent

**Affiliations:** School of Life Sciences, University of Dundee, Dundee DD1 5EH, Scotland, UK; ^†^​ Present address: Oxford BioMedica, Windrush Court, Transport Way, Oxford OX4 6LT, UK.; ^‡^​ Present address: Department of Life Sciences, Imperial College London, South Kensington, London SW7 2AZ, UK.; ^§^​ Present address: Institute of Biology/Microbiology, Martin Luther University Halle-Wittenberg, Kurt-Mothes-Str. 3, 06120 Halle (Saale), Germany.

**Keywords:** metabolic engineering, genetic engineering, fermentation, bio-hydrogen, hydrogenase, pyruvate :: ferredoxin oxidoreductase

## Abstract

*Escherichia coli* produces enzymes dedicated to hydrogen metabolism under anaerobic conditions. In particular, a formate hydrogenlyase (FHL) enzyme is responsible for the majority of hydrogen gas produced under fermentative conditions. FHL comprises a formate dehydrogenase (encoded by *fdhF*) linked directly to [NiFe]-hydrogenase-3 (Hyd-3), and formate is the only natural substrate known for proton reduction by this hydrogenase. In this work, the possibility of engineering an alternative electron donor for hydrogen production has been explored. Rational design and genetic engineering led to the construction of a fusion between *Thermotoga maritima* ferredoxin (Fd) and Hyd-3. The Fd-Hyd-3 fusion was found to evolve hydrogen when co-produced with *T. maritima* pyruvate :: ferredoxin oxidoreductase (PFOR), which links pyruvate oxidation to the reduction of ferredoxin. Analysis of the key organic acids produced during fermentation suggested that the PFOR/Fd-Hyd-3 fusion system successfully diverted pyruvate onto a new pathway towards hydrogen production.

## Full-Text

Under anaerobic fermentative conditions, *Escherichia coli* performs formate-dependent hydrogen production [1]. This is catalysed by the formate hydrogenlyase (FHL) complex [2–4], which is a membrane-bound enzyme comprising [NiFe]-hydrogenase-3 (Hyd-3) and a formate dehydrogenase component encoded by *fdhF* [5]. FdhF is loosely attached to Hyd-3 via the HycB protein, which itself contains four [4Fe-4S] clusters [2]. The *E. coli* Hyd-3 isoenzyme is unusual for a nickel-containing hydrogenase as it is apparently tuned towards proton reduction rather than H_2_ oxidation [2]. However, this makes Hyd-3 an attractive candidate for engineering hydrogen production activity.

FHL subunits share sequence similarity with the membrane-bound hydrogenases (MBH) from, for example, *Pyrococcus furiosus* [6]. The electron donor for *P. furiosus* MBH is not a formate but a reduced ferredoxin [6, 7], probably generated by pyruvate :: ferredoxin oxidoreductase (PFOR) [8]. PFOR is a cytoplasmic enzyme that oxidizes pyruvate to generate CO_2_, acetyl-CoA, and reduced ferredoxin with a midpoint potential (*E*_m_) estimated at –500 mV [9].

**Fig. 1.**
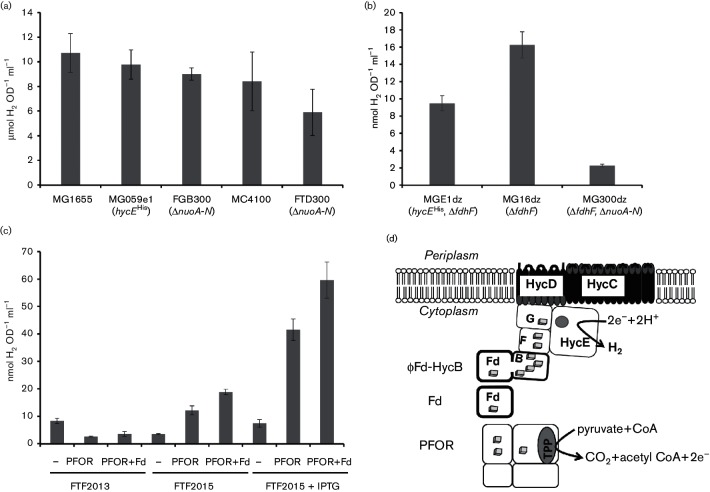
A fusion between ferredoxin and Hyd-3 produces hydrogen *in vivo* in the presence of pyruvate-ferredoxin oxidoreductase (PFOR). (a) The parental strains, MG1655 and MC4100, together with derivatives lacking the *nuo* operon encoding NADH dehydrogenase (Δ*nuoA-N*) MG16dZ and FTD300, and the strain MG059e1 (as MG1655, *hycE*^His^), were grown anaerobically in M9 medium supplemented with 0.8 % (w/v) glucose for 24 h after which the OD_600_ was measured and the H_2_ content in the headspace quantified by gas chromatography. Error bars represent sem (*n*=3). (b) Strains carrying Δ*fdhF* deletions were analysed in an identical manner to those described in panel (a); however, the data are plotted separately as the values are 1000 times lower. (c) Strains FTF2013 (φ*fd-hycB*) and FTF2015 (φ*fd-hycB* under control of the T5 promoter) were transformed with pUNI-PROM, pUNI-Tm-POR (encoding *T. maritima* PFOR) or pUNI-Tm-Fd-POR (encoding *T. maritima* PFOR and ferredoxin). The FTF2015 strain also carries pREP4 encoding LacI. Anaerobic M9 medium with 0.8 % (w/v) glucose, 0.2 % (w/v) casamino acids, plus 1 mM IPTG (final concentration) where indicated, was used. Cultures were incubated for 24 h at 37 °C. (d) Depiction of the complete PFOR/φFd-Hyd-3 system activated in *E. coli*.

In this work, pyruvate was explored as an alternative non-natural substrate for H_2_ production from *E. coli* Hyd-3. A rational design approach was taken to covalently attach the ferredoxin from *Thermotoga maritima* to Hyd-3 via the HycB subunit. *T. maritima* Fd and PFOR plasmids were readily available [10]. To begin, strains were constructed where the natural electron donor enzyme for FHL, FdhF, was genetically removed (Table 1) using an available Δ*fdhF* allele [11]. *In vivo* hydrogen production assays involved measuring the accumulation of H_2_ in the headspace (10 ml) of anaerobic cultures (5 ml) in Hungate tubes containing 0.8 % (w/v) glucose. Following incubation at 37 °C, H_2_ was quantified using gas chromatography (Shimadzu GC-2014) with N_2_ as carrier (25 ml min^−1^). The *fdhF* mutation resulted in a reduction in H_2_-evolution activity of 1000 times compared to the original parent strain (Fig. 1a, b). This *fdhF* mutant phenotype was repeated in a strain carrying a chromosomal *hycE*^His^ allele (Table 1, Fig. 1b).

**Table 1.** Strains and plasmids used or constructed in this study

Strain	Relevant genotype/description	Source
MC4100	*E. coli* K-12: F-, *araD139*, Δ(*argF-lac*)U169, *ptsF25*, *deoC1*, *relA1*, *flbB5301*, *rspL150*	[22]
FTD300	As MC4100, Δ*nuoA-N *:: Apra^R^	This work
MG1655	*E. coli* K-12: F-, λ-, *ilvG*, *rfb-50*, *rph-1*	[23]
FGB300	As MG1655, Δ*nuoA-N *:: Apra^R^	This work
MG16dZ	As MG1655, Δ*fdhF*	This work
MG300dZ	As MG1655, Δ*fdhF*, Δ*nuoA-N *:: Apra^R^	This work
MG059e1	As MG1655, *hycE*^His^	[2]
MGE1dZ	As MG1655, *hycE*^His^, Δ*fdhF*	[11]
FTF2013	As MGE1dZ, Δ*nuoA-N *:: Apra^R^, Δ*hycAB*::φ*fd-hycB*	This work
FTF2015	As MGE1dZ, Δ*nuoA-N *:: Apra^R^, Δ*hycAB *:: φ*fd-hycB*, P_T5_ φ*fd-hycB*	This work
**Plasmids**		
pREP4	*lacI*^+^ (Kan^R^)	Roche
pUNI-PROM	A pT7.5 derivative carrying 103 bp *E. coli tatA* promoter (Amp^R^)	[24]
pUNI-Tm-POR	As pUNI-PROM with *T. maritima* PFOR operon (Amp^R^)	[10]
pUNI-Tm-Fd-POR	As pUNI-PROM encoding *T. maritima* Fd and PFOR (Amp^R^)	[10]

FHL subunits share similarity with the respiratory NADH dehydrogenase encoded by *nuoA-N* [3, 12, 13]. A Δ*nuoA-N* allele, marked with apramycin resistance from pIJ773 [14], had no effect on the ability of *E. coli* FGB300 or FTD300 (Table 1) to grow under fermentative conditions or the amount of H_2_ produced (Fig. 1a). Next, Δ*fdhF* and Δ*nuoA-N* alleles were combined in a single strain (MG300dZ) and the double deletion was found to reduce the residual H_2_ production further still (Fig. 1b). It is therefore possible that the very low levels of residual H_2_ produced in the *fdhF* mutants results from reversed electron transport through Hyd-2 [15].

Next, a Δ*hycA *:: φ*fd-hycB* allele was generated that encoded a fusion of *T. maritima* Fd to HycB via an HA epitope tag. Also, to upregulate expression of this fusion, the synthetic T5 promoter, *lac* operator and ribosome binding site from strain FZBup [11] was included to give a Δ*hycAB *:: P_T5_φ*fd-hycB* allele. Two strains, FTF2013 and FTF2015, were constructed (Table 1) and *in vivo* H_2_ evolution activity quantified (Fig. 1c). The FTF2013 and FTF2015[pREP4] strains were transformed with pUNI-PROM (empty control vector), pUNI-Tm-POR (encoding *T. maritima* PFOR) or pUNI-Tm-Fd-POR (encoding *T. maritima* PFOR and Fd) then grown at 37 °C for 24 h in anaerobic Hungate tubes containing 5 ml M9 medium supplemented with 0.8 % (w/v) glucose and 0.2 % (w/v) casamino acids. The FTF2013 strain produced H_2_ at basal levels regardless of the presence of plasmids (Fig. 1c). This basal level was mirrored in the FTF2015[pREP4]/pUNI-PROM strain (Fig. 1c). However, when the PFOR plasmid was introduced into FTF2015[pREP4] hydrogen, evolution increased to >40 nmol H_2_ OD^−1^ ml^−1^ (Fig. 1c). Moreover, the vector encoding both PFOR and extra Fd induced H_2_ production to a maximal level of >60 nmol H_2_ OD^−1^ ml^−1^ in the presence of IPTG (Fig. 1c).

**Fig. 2.**
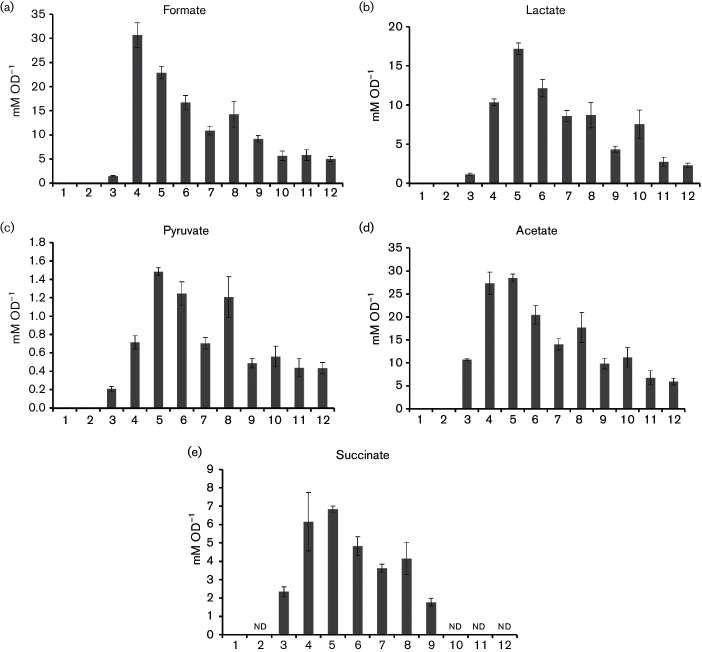
The influence of the Fd-Hyd-3 fusion and PFOR on fermentation products. FTF2013 (φ*fd-hycB*) and FTF2015 (φ*fd-hycB* under control of the T5 promoter) were each transformed with pUNI-PROM, pUNI-Tm-POR (encoding *T. maritima* PFOR) or pUNI-Tm-Fd-POR (encoding *T. maritima* PFOR and ferredoxin). FTF2015 also carries pREP4. Cultures were grown anaerobically in 16 ml LB plus 0.8 % (w/v) glucose and 1 mM IPTG (final), when required, at 37 °C for 24 h. The spent fermentation broth was analysed by HPLC by loading 5 µl on an Aminex HPX-87H organic acid column at 0.5 ml min^−1^ and 55 °C and monitoring absorbance at 210 nm. Organic acid standard curves were used all with R^2^ values greater than 99.90 %. Peaks corresponding to the retention times of (a) formate, (b) lactate, (c) pyruvate, (d) acetate, and (e) succinate were quantified and data normalized to original OD_600_. Error bars represent sem (*n*=3). Note that succinate could not be confidently determined (nd) in samples containing IPTG. Lane 1, virgin LB medium only; lane 2, virgin LB medium + IPTG; lane 3, MC4100 positive control; lane 4, FTF2013 + pUNI-PROM; lane 5, FTF2013 + PFOR; lane 6, FTF2013 + PFOR + Fd; lane 7, FTF2015 + pUNI-PROM; lane 8, FTF2015 + PFOR; lane 9, FTF2015 + PFOR + Fd; lane 10, FTF2015 + pUNI-PROM + IPTG; lane 11, FTF2015 + PFOR + IPTG; and lane 12, FTF2015 + PFOR + Fd + IPTG.

The levels of the most common organic acids produced during mixed-acid fermentation were investigated for strains producing active Fd-Hyd-3/PFOR (Fig. 2). Strains were grown for 24 h in 16 ml LB medium supplemented with 0.8 % (w/v) glucose. Culture supernatants were then passed through a 0.2 µm filter and analysed with an Aminex HPX-87H organic-acid column at 55 °C and 0.5 ml min^−1^. Organic acids were detected by A_210 nm_ and compared to standard curves. Representative HPLC traces are shown in Fig. S1 (available in the online Supplementary Material). The starting concentration of glucose added to the rich medium was 44 mM d-glucose and, under the growth conditions chosen, the MC4100 FHL-positive strain produced 1.5 mM OD_600_ ^−1^ of formate (Fig. 2a) compared with 30.6 mM OD_600_ ^−1^ for the FTF2013/pUNI-PROM strain (inactive for FHL). Importantly, when the PFOR, Fd and Fd-Hyd-3 system is produced at its maximum level, the extracellular formate was observed to drop back to 5.7 mM OD_600_ ^−1^, which is indicative of pyruvate being directed away from the endogenous pyruvate formatelyase (PFL) enzyme to the fusion protein.

Extracellular lactate levels were found to be high in FTF2013 (Fig. 2b). This may mean that the higher formate levels (Fig. 2a) are inhibiting PFL leading to an accumulation of pyruvate and thus extra substrate for lactate dehydrogenase. Indeed, pyruvate can be detected in the growth medium (Fig. 2c) and its level does follow that of formate and lactate in the FTF2013 mutant strains (Fig. 2c). Although pyruvate would not normally be located outside the cell, *E. coli* is known to possess a pyruvate exporter to balance metabolite levels [16], and so any extracellular pyruvate levels may also correlate somewhat with that inside the cell cytoplasm. Importantly, in all cases, when the PFOR, Fd and Fd-Hyd-3 system is maximally produced (FTF2015/pUNI-Tm-Fd-POR + IPTG), the balance of pyruvate/lactate/formate returns to the low levels seen in the FHL-positive strain (Fig. 2c).

Normally, acetate levels are linked to that of acetyl CoA via phosphate acetyltransferase and acetate kinase. The observed increase in extracellular acetate (Fig. 2d) may mean a concomitant increase in cytoplasmic acetyl CoA, which is normally competed for by the AdhE-dependent ethanol production pathway. This is feasible and could be a consequence of the increased activity of the NADH-dependent lactate dehydrogenase already noted for these strains, which would reduce the requirement for AdhE to recycle NAD^+^ and allow acetyl CoA to be used for ATP and acetate production instead.

Together, these data demonstrate the successful re-purposing of *E. coli* Hyd-3 to accept electrons from a new substrate: reduced ferredoxin linked to pyruvate :: ferredoxin oxidoreductase. Examples of native [NiFe]-hydrogenase :: ferredoxin interactions are not common; however, *Synechocystis* sp. PCC 6803 does contain such a system [17]. Physical tethering of a ferredoxin to an [FeFe]-hydrogenase, as opposed to a [NiFe]-hydrogenase, from *Chlamydomonas reinhardtii* showed that photosystem I could be coupled directly to H_2_ production [18, 19]. Similarly, photosynthesis-linked ferredoxins have been fused to cytochromes P450 [20]. Functional fusion of a ferredoxin to [NiFe]-hydrogenases has resulted in some activity *in vitro* [21]; however, the φFd-Hyd-3 enzyme described in these experiments is one new example of a functional fusion that is active in the living cell.

## Supplementary Data

200 Supplementary File 1
