# Supplementary material for: Expanding the substrates for a bacterial hydrogenlyase reaction
Source: Microbiology (Reading). 2017 May 10;163(5):649–53. doi: 10.1099/mic.0.000471 (PMC5817251; doi:10.1099/mic.0.000471)
Supplement: Supplementary File 1 [file mic-163-649-s001.pdf]

Expanding the substrates for a bacterial hydrogenlyase reaction.

Ciaran M. Lamont, Ciarán L. Kelly, Constanze Pinske, Grant Buchanan, Tracy Palmer, Frank Sargent

SUPPLEMENTARY INFORMATION

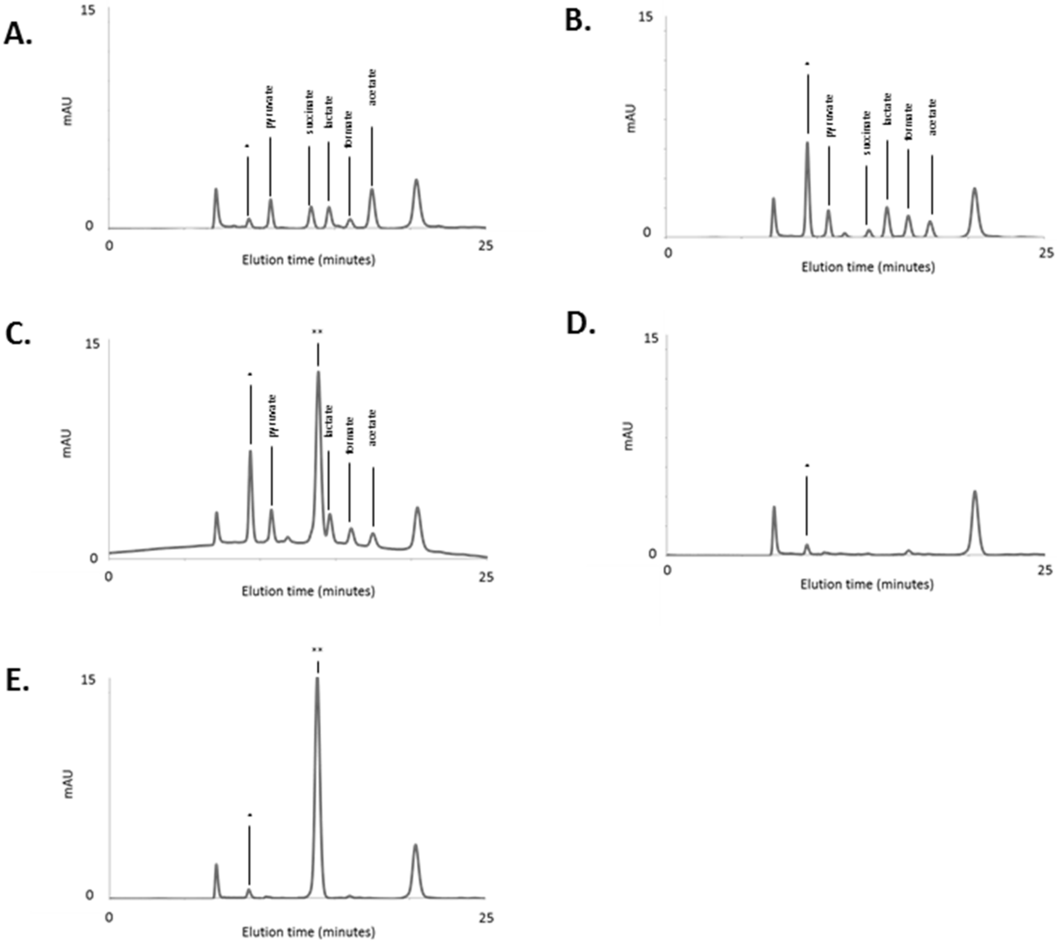

Supp Figure S1: Representative HPLC elution profiles to show organic acid content in spent fermentation broth.

FTF21013 and FTF2015 were each transformed with pUNI-PROM, pUNI-Tm-POR or pUNI-Tm-Fd-POR, before being used to inoculate Universals filled with 16 ml LB + 0.8 % (w/v) glucose were inoculated. Additional cultures were set up for the FTF2015 samples as before but also containing 1 mM IPTG. After the cultures were subject to anaerobic growth at 37 ^{o}C for 24 hours, OD_{600} was measured before cells were pelleted by centrifugation at 4000 rpm. The supernatant was passed through a 0.2 µm syringe filter and analysed by HPLC. For HPLC analysis, 5 µl of fermentation broth was separated at 0.5 ml min^{-1} and 55 °C using a Dionex UltiMate 3000 HPLC system, fitted with an Aminex HPX-87H organic acid column, with absorbance monitored at 210 nm. Organic acid standards were used all with R^2 values greater than 99.90%. Peaks with elution times corresponding to these elution times have been identified. Traces are numbered as follows: **A**: MC4100 + pUNI-PROM; **B**: FTF2015 + pUNI-Tm-Fd-POR; **C**: FTF2015 + pUNI-Tm-Fd-POR + IPTG; **D**: virgin LB media; **E**: virgin LB media + IPTG. Two unidentified peaks of interest are marked by '*' and '**'.
